# Experience of Occupations among People Living with a Personality Disorder

**DOI:** 10.1155/2019/9030897

**Published:** 2019-03-27

**Authors:** Olivier Potvin, Catherine Vallée, Nadine Larivière

**Affiliations:** ^1^Institut de Santé Mentale de Québec, Centre Intégré Universitaire de Santé et Services Sociaux de la Capitale Nationale, Québec, Canada; ^2^Université Laval, Rehabilitation Department, Québec, Québec, Canada; ^3^Université de Sherbrooke, School of Rehabilitation, Sherbrooke, Québec, Canada

## Abstract

**Introduction:**

Personality disorders are common mental health disorders, with an estimated lifetime prevalence of 4 to 15%. People living with personality disorders are extensively seeking mental health services, yet few papers focus on their unique occupational needs or effective rehabilitation interventions that may alleviate the occupational issues they face. Occupational therapists are encouraged to support engagement in socially valued occupations, while preventing engagement in damaging ones, despite a lack of evidence on the meaning and the lived experiences of people.

**Objectives:**

This paper describes the meaning attributed by people living with personality disorders to their main occupations and the underlying needs they strive to fulfill through occupational engagement, whether or not these occupations are sanctioned.

**Methods:**

This exploratory study rests on a descriptive interpretative methodology. The participants were ten men and women, aged between 18 and 35 years old and living with a Cluster B personality disorder. A semistructured interview guide allowed participants to build narratives on occupations that are important to them and discuss how these occupations shape their identity. A thematic content analysis fostered the development of a coding structure that reflected a first-account perspective.

**Results:**

The narratives provided by the participants depict a variety of meaningful occupations, many of which are socially disapproved. Many of these occupations serve as a coping strategy to deal with distressing situations, to connect with others who share similar life experiences, or to reestablish a fragile sense of control. Other occupations are socially disapproved due to the overinvestment of the participants' commitment. While participants described how this overinvestment allowed them to control destructive impulses, significant others perceived it as counterproductive and unnecessary. Participants perceived self-care occupations as painful and tedious chores or meaningless occupations. Engaging in productive occupations allowed some participants to gain recognition or to identify their competencies, but also confirmed their differences, creating some form of alienation or marginalisation.

**Conclusion:**

This exploratory study invites clinicians and researchers to develop a more responsive understanding of occupational engagement for this population. The results highlight the importance of situating occupations in their context, while endorsing a first-account perspective, to better understand the forces that shape occupational engagement. Ultimately, occupational therapists should critically appraise their assumptions around healthy and unsanctioned occupations, in order to respond with sensitivity to the needs and experience of their clients, without perpetuating the marginalisation and discrimination they face.

## 1. Introduction

The lifetime prevalence of mental disorders in Canada is estimated at 20% [1]. Of these, personality disorders (PD) have a lifetime prevalence between 4 and 15% [2, 3]. This population represents 50% of the clients served by mental health services [3]. In the province of Quebec, 2.5% of men and 3% of women live with PD. Due to a higher rate of suicide, living with a PD may reduce their life expectancy by 9 to 13 years [4]. Cluster B personality disorders are the most common [5]. This cluster includes borderline, narcissistic, antisocial, and histrionic PD. Borderline PD is a pattern of instability in self-image, interpersonal relationships, impulsivity, and affects. Those who live with narcissistic PD manifest grandiosity, an excessive need for admiration, and a lack of empathy. Antisocial PD is a pattern of disregard for social norms and violation of the rights of others. Finally, histrionic PD is characterized by constant attention-seeking, emotional overreaction, and suggestibility. However, these diagnoses often cooccur with each other [5–7].

People living with these disorders face enduring and pervasive difficulties in the following areas: cognition (ways of perceiving and interpreting themselves, others, and events), affectivity (range, intensity, lability, and appropriateness of emotional response), and interpersonal functioning and impulse control, leading to an impairment in social and occupational functioning [6]. Use of maladaptive defense mechanisms such as splitting, denial, projection, rationalisation, omnipotence, and acting out also affects functioning in daily activities [7].

These difficulties affect their quality of life, while compromising engagement in meaningful occupations. Meaningful occupations refer here to all activities considered important by the individual, aimed at fulfilling fundamental needs (e.g., autonomy, competence, and belonging) and which may bring meaning to life [8–10]. However, people living with PD find little meaning and satisfaction in their occupations and their daily routine [11–13]. In addition, many engage in occupations that may potentially be harmful [14, 15]. These occupations are viewed as unacceptable, unhealthy, illegal, or immoral [16]. Rehabilitation interventions for this population aim at supporting engagement in occupations that are socially sanctioned [17], while reducing engagement in potentially damaging occupations [18]. Also, scholarly literature suggests that identity and occupational engagement are inextricably linked [19, 20]. Occupational engagement can provide opportunities for developing identity, meaning, and a sense of purpose, while exercising competencies and contributing to the community [21, 22]. This concept can also be defined as full participation in occupations for purposes of doing what one needs and wants to do, being, becoming who one desires to be, and belonging, through shared occupations in communities (Hammell, 2014; Wilcock, 1999).

People living with a Cluster B PD struggle in maintaining meaningful relationships and affiliation; nonetheless, their active quest for acceptance or recognition from others, both through their interactions and their occupations, is well documented. As suggested by some authors, one can also find meaning and shape identity through potentially harmful and antisocial occupations [22]. Yet, although identity disturbances may be at the core of Cluster B PD, there is little empirical evidence on the relationship between occupational engagement and identity, among this population.

The overall purpose of this study consisted of exploring how occupational engagement, as experienced by people living with a Cluster B PD, shapes their self-identity. More specifically, this first objective aimed at exploring how occupations allow people living with a PD to fulfill their needs and how they contribute to their self-identity, either positively or not.

However, a better understanding of the nature of the occupational engagement of people living with a PD requires a contextualised assessment of their occupational experiences [23], as well as some considerations for the underlying needs they are striving to fulfill. As outlined by some authors, occupations related to well-being are not positive or negative, but neutral. They may all have positive or negative impacts, depending on context [24, 25]. As suggested by Hammell [26], the categorisation of occupations may restrict our understanding and purpose of occupations, while marginalising groups whose participation falls outside of this categorisation. The imposition of categories of occupations is likely to obscure the vision and perception of minorities for the benefit of the majority. Moreover, unsanctioned occupations that are often associated with Cluster B PD, such as self-harm, substance use, or acting out, may hold a different meaning and purpose for those who engage in them [27]. Adopting an inclusive definition of occupations, including those that fail to promote growth, social interaction, productivity, or well-being, may allow occupational therapists to develop a deeper and contextualised understanding of occupations [28].

Therefore, the second objective of the study, as reported in this paper, consisted of describing the purpose and functions of daily or important occupations, as experienced by people living with a Cluster B PD, regardless if these occupations are sanctioned or not.

## 2. Methods

### 2.1. Study Design

This exploratory study is based on a descriptive interpretative methodology [29]. By using an inductive approach, this method allows us to define a phenomenon by identifying its characteristics and components from the participant's perspective [29–31], which fits with the purpose of this paper. The use of such methodology also enables the contextualisation of the participant's occupations in order to accurately report their meaning [32].

### 2.2. Sampling

Men and women aged between 18 and 35 years old (*n* = 10), living with a moderate to severe Cluster B PD, recruited using a purposive sampling strategy with maximal variation, took part in the study [33]. Cluster B PD are often misdiagnosed; people who manifest disturbing behaviours, such as acting out, persistent parasuicidal behaviours, or recurrent suicidal conducts, may be wrongfully diagnosed as living with a Cluster B PD. In order to reduce the risk with respect to the validity of the study, recruitment took place in a highly renowned specialised clinic in the province of (name of the province), which exclusively serves this population. Occupational therapists are members of this specialised team. Clinicians who work at this clinic receive extensive training on differential diagnosis, based on the DMS-5, and regarding treatment. Therefore, the clinic only accepts referrals from psychiatrists when current mental health services are ineffective or insufficient. Based on these premises (since it is a criteria for admission to the clinic), it is safe to assert that the participants are indeed living with a moderate to severe Cluster B PD. Most clients served at this facility struggle to keep a job or maintain meaningful relationships and often display self-destructive or harmful behaviours.

Inclusion criteria for selecting participants were (1) to live in the city of (name of the city); (2) to have received mental health services over the last year; (3) to be able to provide free consent; (4) to express themselves in French; and (5) to be aged between 18 and 35 years old. The decision to circumscribe the sample to young adults is explained by (1) the importance of recruiting participants who are facing similar milestones in developing their identity; (2) their prevalence among people living with PD who seek mental health services; and (3) the higher level of interference of the symptoms on their functioning and the impact of this interference on the prognosis. The exclusion criteria were (1) to present a comorbidity of psychotic disorder or a neurodevelopmental disorder and (2) to be admitted in a psychiatric emergency, crisis centre, or day hospital during the data collection period. By adding the last criteria, the research team acknowledges that potential participants may struggle to barely survive and may not be able to cognitively and emotionally engage in a research project.

### 2.3. Data Collection

This study was approved by the Research Ethics Committee of the (name of the Institute) Research Centre. All members of the research team remained blind to the clinical diagnosis of the participants and to the interventions they received. The data collection was conducted over a one-year period between May 2017 and May 2018. Recruitment took place in a specialised outpatient clinic, dedicated to the treatment and rehabilitation of people living with Cluster B personality disorders, upon the clients' admittance to the clinic. Clinical team coordinators identified potential candidates and certified that the selection criteria were met. Once potential candidates approved, the first author made an appointment and solicited an informed consent.

The first author, who also has extensive clinical experience working with this clientele, conducted all the interviews. A semistructured interview guide allowed participants to develop narratives describing meaningful or personally important occupations. This interview guide was previously pretested with two clients of (name of the clinic) to verify the clarity and sequence of the questions as well as the understanding of the topic to be explored. Participants initially selected two important or meaningful occupations in their lives that they felt comfortable discussing. By means of the interview, they shared their perspective on these occupations, their meaning, function, or purpose, as well as how they engage in these occupations. The interview guide assisted participants in offering narratives that would detail how these activities unfolded and the spatial, temporal, and social contexts in which they took place.

In order to meet both study objectives, participants were subsequently invited to describe in a similar way, occupations that (1) made them feel competent; (2) gave them a sense of control; (3) gave them a sense of accomplishment; and (4) reflected who they are. This process allowed each participant to provide several narratives about their occupations.

### 2.4. Data Analysis

Following the interview, participants were called by the first author to validate the initial analysis of the narratives before the research team undertook the formal analysis process. This allowed participants to situate their experience, to further contextualise their narratives and confirm the trustworthiness of the initial understanding of the research team.

Recruitment took place until no new codes emerged from the analysis of data; in fact, all codes regarding occupational experiences, meaning, structure, or functions of occupations remained stable after eight interviews. Two additional interviews were conducted in order to obtain the saturation of the first objective (contribution of occupational engagement to identity).

A thematic content analysis was conducted, using the QDA Miner software, to describe the phenomenon and identify its specific characteristics [34, 35]. Based on the content of the interviews, inductive analysis was used to highlight the relevant and consistent categories that describe how engagement in occupations and their meaning shape the identity of the participants [36, 37]. As suggested by Miles and Huberman [38], the analysis included three concurrent streams of activities: (1) aggregation of data (through the refinement and condensation of the coding tree); (2) presentation of characteristics and their interconnections; and (3) development and verification of conclusions.

Each narrative was coded independently, then compared and peer-reviewed within the research team. The members of the research team defined new codes, as they emerged from the inductive analysis, while challenging their perspective and adopting a reflective stance. The research team ensured that the different codes remained of the first-account perspectives of participants and the context in which their occupations took place. In a second phase, the research team, through iterative processes, developed an elaborated description of each central theme, where the different categories of occupations were described and situated. There was a substantive effort to distinguish the categories and clarify their characteristics [29]. Throughout this reduction phase, constant comparisons within and across narratives allowed to examine emerging hypotheses and refine the analytical process [38, 39]. The entire process was peer-reviewed for the purpose of enhancing the credibility of the study [40] and challenging possible assumptions. Finally, a methodological journal integrated into the software served as a logbook to record observations, impressions, hypotheses, and questions raised in the field. The definitions and modifications of the various codes were also documented.

## 3. Results

### 3.1. Description of Participants

Five men and five women participated in the study. Two had children and most lived alone. As previously discussed, the research team did not request the formal diagnoses from the treatment team but as indicated in Table 1, most participants reported living with a PD (*n* = 8), without specifying a clear diagnosis, except one participant who mentioned having borderline PD.

At the time of the interview, three participants were workers while four were students. Most participants described their struggles as they tried to keep or invest themselves in their productive occupations. Some participants refused to answer certain sociodemographic questions, while contributing to all the other aspects of the data collection process. Although the socioeconomic status of all participants remains unknown, 60% of participants disclosed that they were living below or close to the poverty line [41]. Globally, the participants self-reported that their PD significantly interfered with their daily functioning.

### 3.2. Description of Occupations

Throughout their narratives, participants mentioned 49 occupations. Of these, they described in depth several meaningful or personally important occupations (*n* = 16), some valued socially, others repudiated, criticised, or disapproved. Their way of framing their occupations seemed primarily influenced by comments of members of their support network and, to a lesser extent, by their own occupational experience. In fact, when asked to describe the meaning of their occupations, the participants tended to focus on the function of their occupations. They extensively described how they were looking for potential outcomes or effects on their mood, sometimes without being fully aware of why they engaged in some occupations. The function or purpose of their occupations was rarely exposed but derived from the effects of their own experiences.

The analysis reveals four types of experiences, with different levels of social approval: (1) when occupations are socially disapproved; (2) when overinvestment/overengagement in occupations is socially disapproved; and (3) when occupations are socially valued.

#### 3.2.1. When Occupations Are Socially Disapproved

The first category refers to occupations that are socially disapproved, often perceived as socially unacceptable, unhealthy, dangerous, immoral, or inappropriate [16]. Some are not sanctioned by society, while the majority are overtly disapproved or criticized by members of the social network of participants. Several participants (*n* = 7) reported engaging in such occupations, as shown in Table 2. Participants and their social network perceived these occupations negatively. Participants often simultaneously engaged in more than one of these occupations.

Participants however engaged in these occupations to meet personal needs. They took a prominent place in their lives since they allowed participants to cope with distress and modulate the PD symptoms. These occupations offered some distraction, an opportunity to distance oneself from harmful situations, and a break from invading thoughts, while allowing participants to focus their attention on a tangible and meaningful objective, as shown by Participant 10 in the following excerpts. (The research team translated the quotes since the participants expressed themselves in French.)

When I play video games, it's often silence in my head. I am focused on the game. (…) I am totally focused. That's why it helps me. The silence in my head and being focused on something I like to do.

It helps me relax. I do not like my work and at some point I become saturated with negative thoughts. In the evening, I smoke pot and it relaxes me. It helps me move on and go to bed. One way to (…) temper my days and reduce the pressure of the work I have to do.

These disapproved occupations sometimes serve as an outlet, providing an excuse to release tension or to ignore the context they are in, as described by one participant who enjoys going out to bars and lashing out at others: “It's liberating. You live your life and the others around you don't really matter anymore. We have fun, we insult who we want. It is pleasant. Feel really liberated” (Participant 9).

The same participant describes how spending most of her time in bars provides a context where disinhibition is accepted and where it is possible to ignore social conventions:

Everybody talks to each other and everybody is drunk. Everyone loves everyone. It is not like that in society. People are real. In bars, if you have something to settle with someone, you can settle it. I saw girls from my graduating class. They were laughing at me. I did not refrain myself from saying what I wanted to say. It made me feel good. I feel better when others hear what I have to say.

Several participants indicated that engaging in such occupations allows them to cope with difficult emotions. For example, Participant 10 who spends most of his time playing video games says: “It helps me rid myself of the accumulated anger. I play to release it and I don't feel it anymore. (It is) a way of letting go of the anger”.

Socially disapproved occupations also restore a sense of control in the lives of some participants. But this feeling of being in control is fragile, supported through a limited number of occupations, sometimes even a single one. Thus, the occupation offers an escape from a distressing or alienating reality, by exercising control elsewhere, as described by Participant 2 who spends most of his time gaming:

I do not feel that I have a lot of control of my life, but for me, playing games on my computer is a way to avoid this feeling. When I play, I feel in control of my actions and I see their impact. I really feel in control of what I do.

For some participants, this need for control must be embodied. Action elicits body sensations and confirms being in control of one's body, movements, and actions into space. Others claim that, in a life filled with uncertainty, they must at least control their bodily functions. For example, Participant 8 explains the importance of restricting her food intake: “There is so much in life that you cannot control. Joys and sorrows appear without asking (…). To offset that, I need to have absolute control (of something). I decided to take control of my diet”.

Socially disapproved occupations allowed participants to deal with challenging interpersonal relationships, including conflicts: “When my parents invite me to dinner, I go outside to smoke and take the opportunity to drink secretly from my flask. I drink when I am on the verge of cracking or lashing out” (Participant 7). The same participant also described, as others did, how using substances was a part of his identity in occupations that contribute to his identity and allow him to closely affiliate with people who share similar life experiences:

Now, it's really fusion. We are inseparable (So drinking is a way to affiliate with somebody, to establish a relationship with someone who is important to you). Yes, and who experiences the same thing. Who is an alcoholic like me.

These occupations offer a venue to assert oneself, to claim one's difference, or demonstrate superiority, as Participant 5 explained about gaming:

In the ratings, I am often at the top. I was among the top 30 players in Quebec. It helps me feel competent. (…) I have a competitive spirit. I want to be the first, no matter what the cost. Perhaps that's why it helps me feel competent. (…). I know there are players that are way better than I. When I meet them, I focus on defeating them.

Finally, Participant 8 noted that some socially valued occupations, such as restricting food intake, are harmful and should be not be sanctioned. She does not understand why it elicits admiration, as it supports her self-destruction:

It is valued in our society. It makes me sick because it's even more difficult. It's hard enough like that. Why are we being told that it's good to be thin and that we must fast? It's already quite complex. I do not need people asking me for advice on losing weight. I have no advice to give, I have been sick for 15 years.

#### 3.2.2. When Overinvestment in an Occupation Is Disapproved

Five participants described experiences where their overinvestment of daily or weekly time in an occupation resulted in this occupation becoming socially disapproved. These narratives described the discomfort and disbelief of participants, as their engagement in these occupations was an object of scrutiny or repetitive negative comments in their social environment and at times, even perceived negatively by the participants themselves. For instance, as indicated in Table 2, one participant described that she could only interact by texting on her cell phone and used it intrusively. Some participants described spending days at the movies or in bed. Even if these occupations are usually socially accepted, the investment of participants was viewed as excessive and criticised by others. This perception was often internalized by participants who acknowledged that their participation was not aligned with social standards.

For these participants, the overengagement in specific occupations was perceived as excessive or even counterproductive, especially by significant others:

I was often scolded. (…) Because I watched too much TV, too many programs, too many movies. I did not do enough of other things. It's positive and extremely negative at the same time. (…) It's not very good for me either. Too many movies and too much technology are not good for me (Participant 3).

Again, Participant 3 relies on these occupations to escape a distressing context or to modulate painful affects:

I could easily spend my entire days at the movie theatre. Besides, that's what I do on my days off. I go to the movies almost every day. (…) It allows me to escape. It allows me to go into another universe, to escape far away from myself.

Another participant sleeps to numb emotional pain and find shelter: “I go to bed and sleep to forget that I'm alive. It makes me feel good because while I'm asleep, I do not feel anything. I am safe away from society and from pain” (Participant 9).

Such an engagement may prevent destructive or harmful behaviors, as Participant 3 says about going to the movies: “That's what helped me stay alive (…) It saved my life at first and it keeps saving me right now”.

Three participants described that they escaped a difficult reality by living a virtual life by means of social networks and digital technologies, at the expense of investing in their “real” social networks. In addition, keeping a virtual distance assisted participants in maintaining connections and relationships with others, without feeling overwhelmed: “I try to talk about it as little as possible: my cell phone is something that takes up a lot of space in my life. I have a virtual life as one might say” (Participant 1).

Presence on social networks makes it possible to exercise influence or to obtain validation from others, despite the relational issues:

I have a Facebook page on which I publish my texts. I write about racism, physics, stars, life elsewhere, the meaning of life, politics. I try to publish an article every few weeks. I want to make people aware of the society in which we live (Participant 10).

In return, this virtual life also challenges existing relationships: “My best friend often says to me: sometimes we do not want to be with you because you always spend all of your time on your cell” (Participant 1).

#### 3.2.3. When Occupations Are Socially Valued

Almost all participants chose to describe socially valued occupations (*n* = 9). Although these occupations are well appraised socially, at times they were negatively experienced by participants, who felt they were further exposed to stigma or excluded. For many, these occupations are marked by normative standards that may exceed their abilities. The challenges faced by the participants when engaging (or not) in these occupations may reinforce a perception of alterity, marginalisation, or exclusion.

I have not worked for a while. I am ashamed of that. Everyone talks about their wages, how they pay their taxes. They talk about what they hear on the radio and I feel a bit out of the game. I am not in the machine and I feel pretty guilty. I avoid all conversations that could lead me to talk about what I do (Participant 6).

The same participant describes how the process to access student accommodations emphasizes limitations and induces more stigma: “Every semester, I have to send a letter to all my teachers in order to identify myself as a disabled person. The teacher then identifies me. I find that difficult. I feel labeled, it makes me feel paranoid”.

Many participants engage in socially valued occupations with the desire to please or impress others. They mention that it is through social recognition that they can somehow acknowledge their own skills and personal worth. Therefore, the meaning and satisfaction derived from these occupations is highly dependent on the appraisal of others, as one participant describes: “It must be fun to volunteer. It has to be valued by others. I find it pleasant to be valued by both the clients and the organizers. (…) They congratulate you, recognise and value you” (Participant 4)

Engaging in such occupations allows some participants to repair past mistakes, as described by Participant 1:

It means that I am somehow useful. It means that I must be a good person, that I am patient and that I love what I do. (…) I have the impression that all the mistakes I have made, that all the nonsense that I have done, no longer exist for a moment because I am doing something constructive that helps people.

Although socially valued occupations can support the participants' self-esteem, they can also lead to suffering and distress. Some participants had constant doubts about their abilities or talents, while maintaining high expectations for themselves. They felt competent only if their actions were positively appraised:

I cannot believe that I can be valued without being highly efficient. I find it hard to imagine that my friends and my teachers could value my performance if I do not get 95%. Why would they still love me if I do not perform as well? (Participant 8).

Some participants therefore made unrestrained efforts to maintain this recognition, which gave rise to overexertion:

I was working on day, evening, and night shifts. I was sometimes working 16 hours a day, 50 hours a week. It killed me. I invested too much time in my job. For too long, I helped others instead of helping myself (Participant 8).

Also, the same participant described being caught up in a downward spiral. On one hand, she acknowledges the risks associated with high expectations, but she nevertheless actively seeks approval and recognition:

I get a lot of compliments for my grades, scholarships, and stuff like that. I know it's not healthy or human to keep this pace, but at the same time, people congratulate me. Why do they congratulate me, if that's not ok?

Socially valued occupations tend to exacerbate relational issues, especially when participants encounter obstacles, face stress, or receive criticism, often accompanied by a fear of failure. These challenges may compromise occupational engagement and satisfaction, as described by this participant: “My old jobs were so frustrating! I felt like I was out of place. I did not feel competent at all. I just felt like an impostor, that I should not be there” (Participant 2).

Therefore, some participants strive to find ways to be in contact with others, while keeping them at a distance:

Socializing is difficult for me (…) I now know why I lose all my relationships. When I volunteer, it's less present. It's not even present at all. When I volunteer, I meet a lot of people that I do not know (Participant 4).

I tend to work alone and my colleagues … They are not really my colleagues, I just work beside them (Participant 2).

Four participants reported engaging in various sports activities. While societal views tend to value these occupations as a way of improving physical and mental health, participants expressed a different perspective. Very few talked of the impact of these activities on their well-being or their health. Most participants described sportive activities as a mean to serve other occupations. For example, many participants explained that they engaged in sports to improve their performance in online games or exams. Sports also provide opportunities for releasing tension or escaping from invasive preoccupations:

It helps me function. It allows me to free myself from my negative thoughts. Otherwise, I am too anxious, too paranoid. It helps to oxygenate my brain (Participant 5).

Some participants used these occupations to transform themselves. As an example, here is what Participant 6 says about swimming: “I'm fine in the water too. I breathe, but I am no longer a human. I feel like a fish. It is as if I am in another form.”

Finally, two participants described activities of daily living since they felt that these occupations should be perceived as important or essential to life. Yet, they were unsatisfactory and meaningless obligations for them. This tension was central in their narratives and appeared to be a major concern for them. They expressed how these occupations are socially taken for granted and how they feel inadequate or different as they experience little intrinsic motivation to engage in these tedious occupations. As Participant 9 pointed out: “It's like … I must do it because it's part of life. I see it as a chore. I should not see this as a chore, but as an essential thing to do in life.”

These occupations are worthwhile when they are carried out to please or obtain some validation from significant others, as Participant 1 explains:

I am someone who seeks approval from people. Taking care of myself is not something that is usual in my daily life. One day, I would like to be able to wake up in the morning and say to myself: I am doing this for myself.

## 4. Discussion

Participants' narratives depict a variety of meaningful occupations, many of which are socially disapproved. As reported in the literature, results indicate that participants often engaged in some socially disapproved occupations as a strategy to cope with difficult affects or relationships. They can sometimes provide a distraction, an outlet, or a possibility of distancing oneself from distressing situations, as suggested in the literature [24, 42]. They may also allow a venue for evading from the perceived constraints or letting go of tension or anger. For some participants, occupations that are less or not valued socially are the vehicle by which they can reestablish a fragile sense of control or affiliate with people who share similar experiences. As Hammell [42] points out, occupations can act as stress modulators; participants indicate that even the occupations that are socially disapproved or so-called “unhealthy” can be a means to achieve well-being, allowing them to cope with stressful events.

Other occupations are socially disapproved due to the excessive time invested by the participants, according to their social network or social norms. Again, this overinvestment can be explained as a coping strategy, used for tolerating distress and escaping a difficult reality, including virtually keeping others at a distance. Although some participants report that they are effective in controlling parasuicidal or suicidal behaviors, such engagement is heavily judged by others, and at times, by themselves. Yet this overinvestment can nevertheless provide a sense of well-being among the participants. The results of this exploratory study support the idea suggested by Doble and Santha [43] which indicated that occupational well-being is derived from the meaning, satisfaction, and underlying needs, rather than the nature or social value of the occupation.

Several participants engage in sports activities. However, they offer a different perspective for the function of their engagement. While the prevalent social discourse on sports insists on satisfaction, health, or well-being, they point out that these activities serve as an outlet for sublimating suffering, managing anxiety, and achieving a sense of well-being through intense bodily sensations, despite the risk of injury, as reported in the literature [24]. Some participants hope that these occupations may contribute to a transformation of themselves or offer the possibility to become invisible, to be like any other individual. Again, this is consistent with the work of Kiepek and Magalhaes [24] who indicate that the purpose of an occupation is not necessarily rational or always in the best interest of the person. Another innovative finding from this exploratory study is that participants engage in sports activities to increase their performance in other occupations that are either socially valued or not.

Few participants shared narratives on daily life occupations, such as self-care or meal preparation. They thought that they were important, yet they were perceived as painful or tedious chores or meaningless occupations. Although occupational therapists consider these socially taken for granted occupations as the foundations for an independent and self-sufficient life [44], they are often a source of boredom and frustration [45]. Participants reported that they engaged in such occupations only to be approved or recognized by others. As suggested by Milbourn et al. [46], these occupations are deemed important because they are at the foundation of companionship and affiliation, and therefore somewhat associated with recovery. It can be argued that just like the general public, people living with a PD engage in self-care activities largely to seek social approval rather than finding intrinsic motivation or meaning.

Nonetheless, most participants described that they also engaged in socially valued occupations to seek approval from their loved ones. It is through social recognition that participants seek to create some stability and a sense of continuity in their lives, despite chaos and uncertainty [46]. Recognition by others may temporarily confirm a fragile sense of competence. Some hope that through their engagement in such socially valued occupations, they may find redemption, repair past mistakes, and develop a decent and more positive perception of themselves.

Socially valued occupations may enact social control mechanisms that encourage compliance with a standard widely endorsed by the majority [16, 44, 45]. Hence, they may have negative or adverse consequences on health and well-being [25, 47]. Some participants tend to overwork and endorse high-performance expectations, at the risk of jeopardising their physical and mental health. Others limit their social interactions or isolate themselves, to avoid facing social issues, conflicts, or criticism. These results echo the work of Dahl et al. [17] who suggest that people living with a borderline PD often avoid social interactions in the workplace or tend to do more than expected to prevent criticism.

Many participants experience stigma through their occupations, whether socially valued or not, which influences their overall occupational engagement. Their occupational choices and the time they devoted to some occupations is the object of scrutiny and disapproval. The very nature of their occupations, their aims, and their potential impacts are often criticised and subjected to social judgments, like other unsanctioned occupations [16, 47]. Engagement in occupations is governed by power dynamics that may contribute to the marginalisation of some minority groups [16]. The participants in the present study attested that the stigma associated with some of their occupations accentuated their feeling of exclusion or inadequacy, as indicated in the literature [47]. Several participants thus perceived socially valued occupations as normative standards that must imperatively be achieved, despite the challenges imposed by the PD [17]. Finally, the results are consistent with the work of several authors who suggest that valuing and excluding certain occupations also obscure a range of occupational opportunities that would better suit the needs of participants [16, 26, 44, 45].

This exploratory study offers a novel perspective on the experience of occupational engagement of people living with a Cluster B PD, while bracketing assumptions on socially valued, disapproved, or unsanctioned occupations. The research team adopted a reflective stance at all stages of the research process. For example, the interview guide was constructed carefully and tested to ensure that the terms used would not reflect social norms and would allow the participants to freely choose the occupations that were personally important to them. To enhance the credibility of the study, the members of the research team exhaustively documented every step of the research process and extensively used peer debriefing to enrich the definitions of codes and concepts, to challenge their assumptions, or to deconstruct potential inferences, while critically reflecting on their analysis and their decisions.

However, the results cannot be generalized to other contexts, given the limited scope of this exploratory study. The sample size is small and might not be representative of the diversity of experiences associated with Cluster B PD. The limited exposure to participants may have limited the research team from developing a stronger rapport or from fully considering the context of the occupations or accessing other relevant experiences, despite the validation of the analysis with the participants. The descriptive interpretative design of this study only provided the perspective of people with a lived experience of a PD: it did not allow critically examining the societal forces that shape social participation in occupations, nor the dynamics of resistance that were expressed through occupations.

Nonetheless, data saturation was reached with respect to (1) the importance of unsanctioned or disapproved occupations as coping strategies; (2) the quest for validation and recognition through engagement in socially valued occupations; (3) the importance of paying attention to the function or the meaning, the subjective experience, and the underlying needs that the participants try to fulfill through occupational engagement, in order to understand the perceived benefits of the occupation; and (4) the possibility that further stigmatisation or marginalisation will take place through occupational engagement.

## 5. Conclusion

As suggested by Kiepek (2018), the results of this exploratory study invite clinicians and researchers to develop a more responsive understanding of occupational engagement for this population. Further research is needed to build empirical evidence around the dissonant perspectives set forth in the participants' narratives, regarding taken-for-granted assumptions about healthy and so-called unhealthy occupations. This paper highlights the importance of situating occupations in their context and considering their purpose, in a people-first perspective. It is imperative to acknowledge how occupations may serve as a coping strategy, even when they are socially disapproved or unsanctioned, in order to develop sensitive and responsive interventions for this clientele. Similarly, occupational therapists must avoid assuming that socially valued or deemed healthy occupations are indeed conducive to health; they may carry unreasonable normative expectations and negatively contribute to people's well-being. Ultimately, occupational therapists need to be critically reflective on how they may encourage or discourage participation in certain occupations to respond with sensitivity to the experience of the people they serve, without perpetuating the marginalisation and discrimination they face.

## Figures and Tables

**Table 1:** Description of participants.

Participants: *n* = 10		
Age	Mean (range)	29.60 (21-35)

Marital status	Single Living with spouse Refused to answer	8 1 1

Education	High school degree not completed High school degree completed College degree not completed College degree completed University degree not completed University degree completed	1 0 3 2 3 1

Main productive occupation	Paid work Studying Volunteering Other (professional gaming) Refuse to answer	3 4 1 1 1

Source of income	Wages Social security Savings/loans Insurance	7 1 1 1

Level of income	$0 to $10,000 $10,000 to $20,000 $40,000 to $50,000 Refuse to answer	3 3 2 2

Self-reported diagnosis	Personality disorder (PD) Borderline PD Refused to answer	8 1 1

Perceived impact of condition on overall daily functioning	Very light Mild Moderate Severe Very severe Refuse to answer	1 1 5 1 1 1

**Table 2:** Distribution of the various types of occupational experiences described by participants.

Types of occupational experience	Number of participants who describe such experiences	Occupations described	Frequency of descriptions
When occupations are socially disapproved	*n* = 7	Gaming extensively	*n* = 5
		Substance use	*n* = 3
		Restricting food intake	*n* = 2
		Seeking conflicts or fights	*n* = 1
		Staying in bars	*n* = 1

When overinvestment is socially disapproved	*n* = 5	Intrusive use of cell phone	*n* = 1
		Overinvestment in social networks	*n* = 3
		Spending days at the movies	*n* = 1
		Taking refuge in sleep	*n* = 1

When occupations are socially valued	*n* = 9	Working	*n* = 4
		Sports activities	*n* = 4
		Studying	*n* = 4
		Taking care of others	*n* = 2
		Volunteering	*n* = 1
		Self-care	*n* = 1
		Meal preparation	*n* = 1

## Data Availability

The qualitative data used to support the findings of this study are included within the article.
